# Persistent gut microbiota dysbiosis and metabolic remodeling after ceftriaxone exposure in mice: a cross-study re-analysis

**DOI:** 10.3389/fcimb.2026.1822390

**Published:** 2026-07-31

**Authors:** Andrew D. Winters, Esha Rudagi, Orena Koka, Mariana Angoa-Perez

**Affiliations:** 1 John D. Dingell Department of Veterans Affairs Medical Center, Wayne State University School of Medicine, Detroit, MI, United States; 2 Troy High School, Troy, MI, United States

**Keywords:** antibiotic dysbiosis, BZINB, ceftriaxone, gut microbiome, host physiology, MaAslin2, PICRUSt2

## Abstract

**Introduction:**

Broad-spectrum antibiotics are known to disrupt the gut microbial environment, which can lead to sustained physiological consequences. Ceftriaxone (CTX) is a β-lactam antibiotic widely used in neuroscience research to enhance expression of the glutamate transporter GLT-1, a key regulator of excitatory neurotransmission in the brain. However, CTX also induces marked alterations in gut microbial composition, yet the consistency and functional consequences of these changes across studies remain poorly defined.

**Methods:**

Here, we conducted a cross-study re-analysis of four independent, publicly available murine 16S rRNA gene sequencing datasets to identify robust microbial and metabolic responses to CTX exposure. Using a bioinformatic pipeline for taxonomic annotation and functional inference, we evaluated microbial diversity, community composition, and predicted functional pathways across studies.

**Results:**

CTX treatment consistently reduced gut microbial diversity and altered community composition relative to controls, with only partial recovery over time. Predicted microbial functions were extensively remodeled, with CTX-exposed communities showing enrichment in pathways related to aromatic compound and amino acid metabolism, amine metabolism, and stress response, whereas control communities retained higher biosynthetic potential.

**Discussion:**

These findings demonstrate that CTX exposure induces persistent, system-level restructuring of the gut microbiome, with potential detriment to host physiology.

## Introduction

The gut microbiome forms a diverse and dynamic ecosystem essential for host digestion, immune regulation, and systemic health (Sommer and Bäckhed, 2013; Belkaid and Hand, 2014). Broad-spectrum antibiotics can profoundly disrupt microbial populations within the gut microbiome, sometimes with lasting consequences (Jernberg et al., 2007; Dethlefsen et al., 2008; Jernberg et al., 2010). Among these, ceftriaxone (CTX), a β-lactam antibiotic, has been shown to profoundly disrupt gut microbial diversity and community structure in murine models, resulting in marked depletion of multiple taxa and selective enrichment of a limited subset (Chakraborty et al., 2018; Zhao et al., 2020; Grégoire et al., 2021; Hong et al., 2024; Winters et al., 2024). Such shifts can alter microbial metabolic and ecological functions with downstream consequences for host physiology (Valdes et al., 2018). However, differences in experimental design, sequencing methodology, and analytical pipelines often weaken cross-study comparability and obscure reproducible microbial patterns.

These microbiome effects take on added importance given that CTX is also widely used in studies of neural function and behavior for its ability to enhance expression of the glutamate transporter GLT-1 in the brain (Rothstein et al., 2005). Yet, the possibility that some of the behavioral effects seen in response to CTX are mediated through gut microbiome alterations remains largely unexplored (Rothstein et al., 2005; Cryan and Dinan, 2012; Foster et al., 2017). Based on 16S rRNA gene sequencing studies in murine models, short-term CTX treatment can induce persistent disruptions in gut microbial diversity and community network complexity, with only partial recovery up to 14 months post-exposure (Cox et al., 2014; Hong et al., 2024). Beyond the gut, CTX-related microbial perturbations have been associated with altered neurobehavioral outcomes, including increased expression of anxiety- and depression-like behaviors, potentially through immune and hypothalamic-pituitary-adrenal axis dysregulation (Diaz Heijtz et al., 2011; Zhao et al., 2020). Collectively, these findings show that CTX-induced dysbiosis can influence multiple physiological systems through changes in microbial metabolites, immune signaling, and neuroendocrine regulation. Variation in animal age, sex, treatment regimen, sequencing platform, and analytical approach (including taxonomic classifiers, e.g., SILVA vs. Greengenes; diversity index metrics, e.g., Shannon vs. Simpson; and feature resolution, operational taxonomic units [OTUs] vs. amplicon sequence variants [ASVs]) complicates cross-study comparisons and hinders identification of robust microbial responses that are consistent across diverse experimental conditions (Knight et al., 2018; Pollock et al., 2018; Regueira-Iglesias et al., 2023).

Re-analysis of publicly available 16S rRNA gene sequencing datasets provides an opportunity to address these questions systematically (Duvallet et al., 2017; Abbas-Egbariya et al., 2022; Cheng et al., 2022; Regueira-Iglesias et al., 2023). In this study, we reanalyzed four datasets (Chakraborty et al., 2018; Grégoire et al., 2021; Hong et al., 2024; Winters et al., 2024) examining the effects of CTX on murine gut microbiome using a standardized bioinformatic workflow to infer microbial metabolic pathways from 16S rRNA gene profiles. This unified approach minimized technical variation across studies and enabled robust evaluation of taxonomic and functional shifts, including the identification of taxa reliably altered by CTX and the broader ecological and metabolic consequences of these changes. By integrating taxonomic alterations with predicted metabolic potential, we defined recurrent patterns of CTX−associated disruption across experimental conditions, encompassing changes in diversity, commensal and opportunistic groups, and community metabolic capacity. This integrative analysis establishes a foundation for future work aimed at determining how CTX−induced perturbations in gut microbial ecology may influence neural and systemic function.

## Materials and methods

### Animals and study selection

Publicly available 16S rRNA gene sequencing datasets involving ceftriaxone treatment in mice were identified through targeted searches of the NCBI Sequence Read Archive (SRA) and associated publications. Because only a small number of murine CTX studies exist, the search yielded four datasets that met the predefined criteria: CTX monotherapy, at least one post−treatment recovery time point, and availability of raw sequencing data with metadata. Studies outside this scope (for example, those using antibiotic combinations or germ−free models) were not considered. All four eligible datasets were included in the analysis (see **Table 1**). Both short− and long−term post−antibiotic sampling intervals were analyzed to capture temporal trajectories of microbial diversity, composition, and predicted metabolic function during recolonization. This selection strategy enabled evaluation of persistent microbial alterations and recovery dynamics across independent murine studies.

**Table 1:** Summary of mouse studies examining ceftriaxone (CTX)-induced gut microbiome alterations across different strains, ages, and treatment regimens.

Study	Strain	Sex	Age (Weeks)	Sample size (Control/CTX)	Days post CTX	16S rRNA region	Key findings	Accession
Chakraborty et al., 2018	C57BL/6	Male	5	41/41	2, 6, 12	V4	Beta diversity shifted by day 4 with decreases in Bacteroidales and Lachnospiraceae and increases in *Lactobacillus* and *Bacteroides*; alpha diversity partially recovered	PRJNA489674
Grégoire et al., 2021	SWISS	Female	6	20/20	3, 7	V4	Alpha diversity decreased significantly after ceftriaxone; beta diversity was markedly altered with distinct trajectories over 7 days, including increases in *Lactobacillus*, Enterobacteriaceae, and *Parabacteroides*.	PRJNA701545
Hong et al., 2024	C57BL/6	Male	4	108/111	22, 52, 82, 112, 142, 172, 202, 232, 262, 292, 322, 352, 382, 412	V4-V5	Alpha diversity decreased acutely after ceftriaxone, partially recovering over 14 months; beta diversity was markedly altered, with transient increases in *Enterococcus*, Pseudomonadota, Desulfobacterota, and Bacteroidota, which gradually normalized.	PRJNA863425
Winters et al., 2024	C57BL/6	Both	7-8	72/84	1, 4, 8, 61, 96	V4	Beta diversity shifted immediately after ceftriaxone, persisting through day 96; acute-phase decreases in Bacteroidota and Actinobacteriota with increases in Firmicutes, *Enterococcus*, and *Bacillales*; alpha diversity decreased acutely; Chao1 remained lower than controls, while Shannon gradually recovered by day 96.	PRJNA1136882

### Raw 16S rRNA data processing and taxonomic annotation

Raw FASTQ files were retrieved from the National Center for Biotechnology Information (NCBI) Sequence Read Archive (SRA) using fasterq-dump -S from sratoolkit version 3.1.0. 16S rRNA gene sequences from four selected studies (Chakraborty et al., 2018; Grégoire et al., 2021; Hong et al., 2024; Winters et al., 2024) were processed with DADA2 version 1.22.0 to generate ASVs, producing merged, denoised, and chimera−free sequences. Sample metadata were compiled and standardized across studies, then imported, along with ASV counts and taxonomic assignments, into R for compatibility with phyloseq version 1.46.0 (Hong et al., 2024). Individual phyloseq objects were created for each BioProject and merged into a single object. Taxa were agglomerated at the genus level, non-bacterial sequences were removed, and select phylum names were updated according to current nomenclature (Proteobacteria: Pseudomonadota, Firmicutes: Bacillota, Cyanobacteria: Cyanobacteriota).

### Alpha diversity

Alpha diversity metrics were computed to evaluate within-sample gut microbial richness, heterogeneity, and evenness. ASV counts were processed using the phyloseq package in R (version 4.5.2). Prior to diversity estimation, samples were agglomerated at the genus level, and taxa with inconsistent annotations were manually curated to ensure consistent taxonomic labels. The dataset was rarefied to 2,392 reads per sample to control for differences in sequencing depth, resulting in a minimum Good’s coverage of 0.987, indicating near-complete sampling of community diversity.

Three alpha diversity indices were calculated using the estimate_richness function in phyloseq: Chao1 richness, Shannon diversity, and Inverse Simpson diversity. Group-level differences were assessed with generalized linear models (GLMs) that included CTX treatment and Time (cube-root transformed) as predictors; Sex was included only as a covariate to adjust for baseline differences in the one dataset containing both sexes. Model significance was evaluated using Type II ANOVA (F-test) via the car package. To assess robustness to study-level heterogeneity, we performed a sensitivity analysis that included BioProject ID as a fixed effect in representative GLMs to evaluate whether CTX effects persisted after accounting for study-specific baselines.

### Beta diversity

Beta diversity was analyzed to assess differences in gut microbial community composition between treatment groups. Genus-level rarefied counts were converted to relative abundances by dividing each sample’s counts by its total reads. Bray-Curtis dissimilarity matrices were calculated using vegan version 2.6.10 (Dixon, 2003). Principal coordinates analysis (PCoA) was performed to ordinate samples based on community dissimilarity, and the first three axes were used for three-dimensional visualization. Interactive 3D plots were generated with plotly version. 4.10.4 (Sievert, 2020) including group-specific 95% confidence ellipsoids and variance-explained percentages for each axis. Temporal effects were explored using the veganvegan envfitfunction to fit sampling time as an environmental vector.

### Differential abundance

Differential abundance of gut microbial taxa was evaluated using Maaslin2 version 1.24.1 (Mallick et al., 2021). Taxon-level counts from rarefied phyloseq objects were used as input, with samples as rows and taxa as columns. Taxa were filtered to retain those present in at least 10% of samples. Sample metadata included sex, treatment group, and cube−root−transformed time as covariates, and post−treatment samples were analyzed separately for comparisons between treatment groups (CTX vs. Control). Counts were normalized using Trimmed Mean of M−values (TMM), and negative binomial regression models were fitted to test for associations between taxa abundances and fixed effects. Reference levels were specified for categorical variables (sex = female; group = Control), and no additional data transformation was applied. Statistical significance was evaluated for each taxon while adjusting for the included covariates.

### Taxon-specific temporal dynamics following antibiotic treatment

To investigate temporal changes in individual taxa following CTX treatment, the top differentially abundant genera identified by Maaslin2 were analyzed using generalized additive models (GAMs). Features were selected based on significance (*q* < 0.01) and effect size (|log_2_ fold difference| ≥ 2), retaining the five most positively and negatively associated taxa.

Predicted abundances from the fitted Maaslin2models were extracted and merged with sample metadata. Cube-root transformed sampling time was used as the independent variable to stabilize temporal trends, while treatment group (CTX vs. Control) served as a categorical predictor. GAMs were fitted for each taxon separately using a smoothing spline s(x, k = 4) to model non-linear temporal dynamics, with 95% confidence intervals displayed. Predicted abundances were visualized to facilitate direct comparison of temporal trajectories between treatment groups.

### Inferred pathway analysis

Functional potential of the gut microbiome was predicted using PICRUSt2 version 2.5.2 (Douglas et al., 2020) with default settings. ASV sequences were placed into the reference 16S phylogeny using place_seqs.py, and EC gene family abundances and 16S rRNA copy numbers (including NSTI scores) were predicted using hsp.py. Metagenome contributions were generated with metagenome_pipeline.py, and MetaCyc pathway abundances were inferred using pathway_pipeline.py with per-sequence abundance and per-sequence function enabled, using the MetaCyc database (Caspi et al., 2020). Final EC and pathway tables were annotated using add_descriptions.py.

modeling Predicted pathway abundances were exported as unstratified tables and merged with sample metadata. Pathways were filtered to include only those with a minimum abundance of 10 and a minimum prevalence of 25% across samples. Differential pathway abundances between treatment groups were assessed using Maaslin2 with TMM normalization and negative binomial regression, including sex, treatment group, and cube-root-transformed time as fixed effects. Statistical significance was evaluated using a false discovery rate threshold of q ≤ 0.05.

### Taxon-pathway associations

To explore associations between microbial taxa and predicted functional pathways, normalized and transformed abundance matrices for genera and pathways were analyzed using IntegrativeBayes version 1.0 (Jiang et al., 2021) to perform Bayesian zero-inflated negative binomial (BZINB) regression modeling. Top features for both taxa and pathways were selected based on significance (q < 0.01) and effect size (|log2 fold-change| ≥ 1) from Maaslin2 results. For each dataset, the 10 features (bacterial taxa or pathways) with the largest absolute coefficients in the Maaslin2 results were retained to define the subset included in subsequent BZINB modeling.

A design matrix representing treatment group (CTX vs. Control) was included as a covariate in the model. The BZINB model was fitted using zinb_w_cov with 100 posterior samples to estimate associations between taxa and pathways, while accounting for excess zeros and overdispersion in the count data.

Posterior mean associations were calculated for all taxon-pathway pairs within these selected features, allowing direct comparison of functional connectivity between taxa and pathways. Differential association matrices were visualized as heatmaps with pathways as rows and taxa as columns, allowing direct comparison of taxon-pathway relationships between treatment groups.

## Results

### Alpha diversity

**Figure 1:**
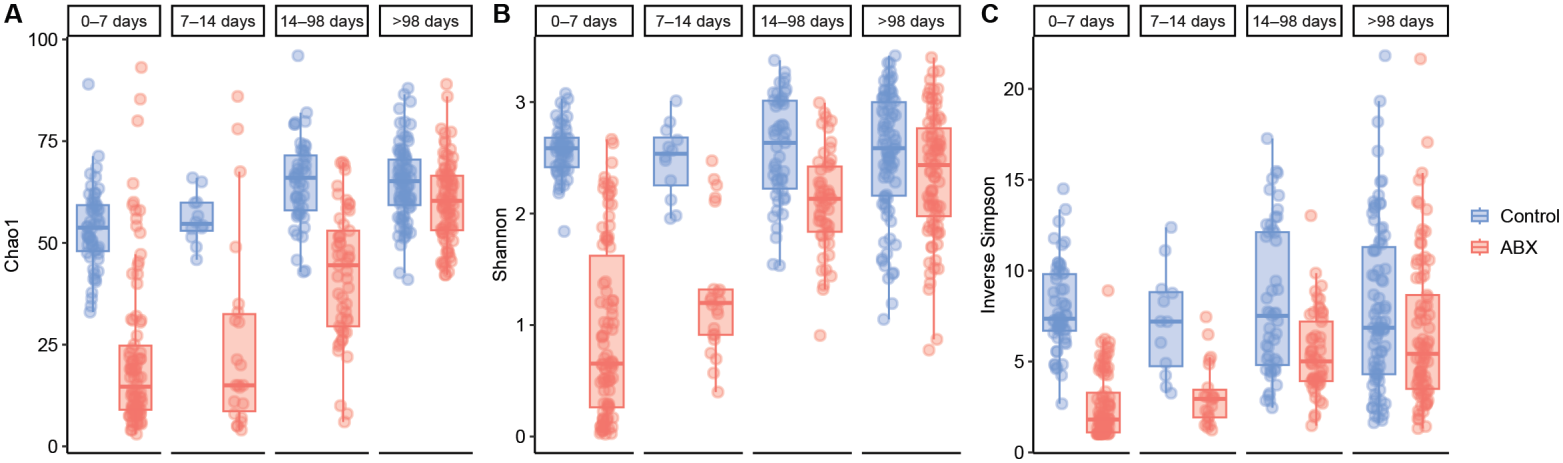
Box-and-whisker plots showing Chao1 richness **(A)**, Shannon diversity **(B)**, and Inverse Simpson diversity **(C)** from fecal 16S rRNA gene profiles of Control and antibiotic-treated (ABX) mice. ABX samples are grouped by time post-exposure (0-7, 7-14, 14-98, and >98 days). Control samples are shown as a single group. Boxes represent the interquartile range with the median indicated by the central line; whiskers extend to 1.5×IQR, and individual samples are overlaid as jittered points.

In the primary GLMs that included CTX treatment, time, and sex, CTX treatment significantly reduced all three alpha−diversity metrics (**Figures 1A–C**). Time was also a significant predictor across models, whereas sex was not. Chao1 richness was significantly reduced by CTX treatment (p < 0.0001), with significant effects of time (p < 0.0001) and a time × treatment interaction (p = 1.2×10^−14^); richness increased over time in all groups. Shannon diversity showed a similar CTX−associated reduction (p < 0.0001), with significant effects of time (p < 0.0001) and a time × treatment interaction (p < 0.0001); Shannon values increased over time across groups. Inverse Simpson diversity was likewise reduced by CTX treatment (p < 0.0001), with significant effects of time (p < 0.0001) and a time × treatment interaction (p = 3.0×^−7^); evenness increased over time in all groups.

To assess robustness to study−level heterogeneity, we performed a sensitivity analysis that repeated these GLMs with BioProject ID included as a fixed effect. BioProject was significant, as expected for multi−study datasets, but its inclusion did not alter the significance or direction of CTX treatment effects for any alpha−diversity metric (all p < 2.2×10^−16^).

### Beta diversity

**Figure 2:**
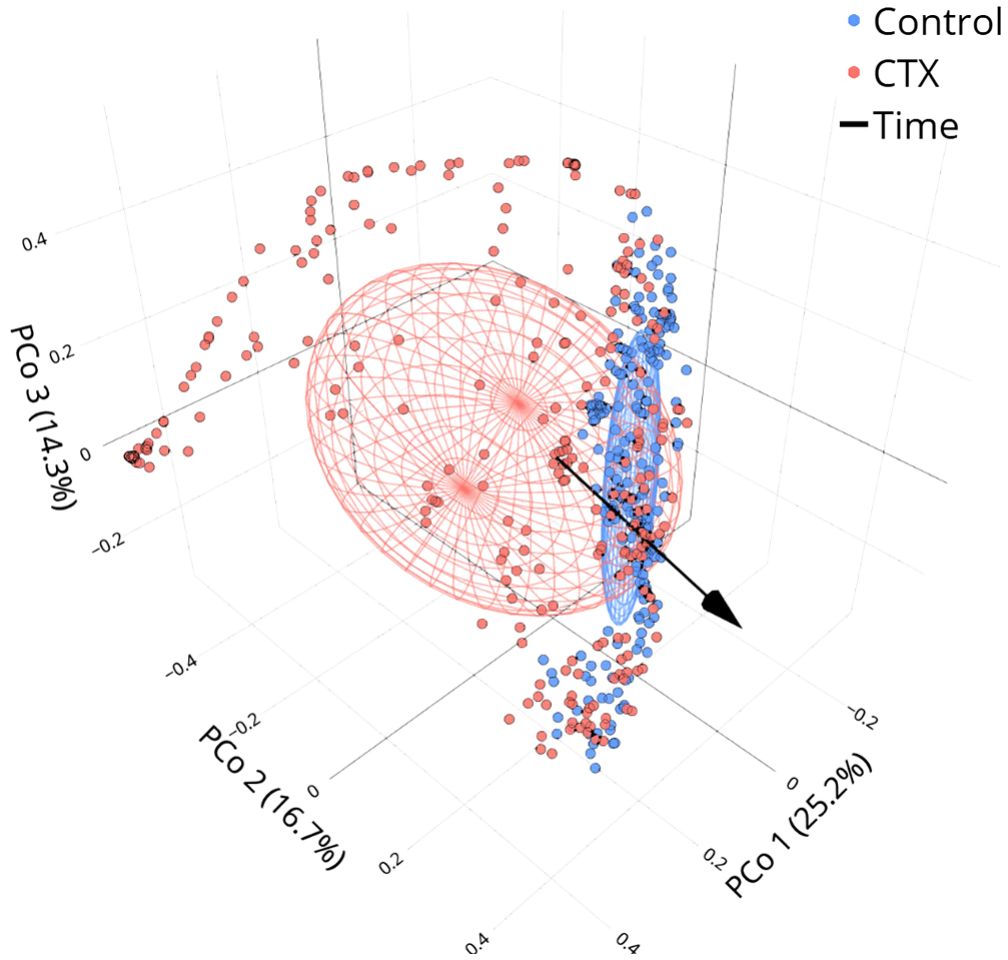
Principal coordinates analysis (PCoA) showing variation in microbial community structure based on Bray-Curtis dissimilarities of fecal 16S rRNA gene profiles from Control and ceftriaxone (CTX)-treated mice. The black arrow represents the fitted temporal gradient, with its direction indicating increasing time.

CTX treatment remained a significant predictor of Bray-Curtis beta diversity after including sex and time in the model (R² = 0.066, p = 0.001). Time (R² = 0.095, p = 0.001) and sex (R² = 0.057, p = 0.001) also explained significant variation in microbial community structure, indicating that each factor contributed independently to community differences. The ordination in **Figure 2** shows a separation of CTX−treated mice compared to controls, consistent with an effect of antibiotic exposure. Samples collected at later time points after stopping CTX cluster closer to the Control group in ordination space compared to earlier time points, reflecting partial recovery of community structure over time. To assess robustness to study−level heterogeneity, we performed a sensitivity analysis including BioProject ID as a fixed effect in the PERMANOVA model. BioProject was significant (R² = 0.094, p = 0.001), as expected for multi−study datasets, but its inclusion did not alter the significance or direction of CTX, time, or sex effects.

To evaluate recovery beyond alpha diversity, we performed ANOSIM within four biologically defined time windows. CTX and control communities were strongly separated during the acute phase (0–7 days; R = 0.439, p = 0.001) and remained significantly distinct during early recovery (7–14 days; R = 0.359, p = 0.001). Separation decreased substantially during the mid−term window (14–98 days; R = 0.1176, p = 0.001), consistent with partial recovery. Notably, in the only study extending beyond 98 days, CTX and control communities remained weakly but significantly distinct (>98 days; R = 0.028, p = 0.014), indicating incomplete long−term convergence despite substantial rebound.

### Differential abundance

**Figure 3:**
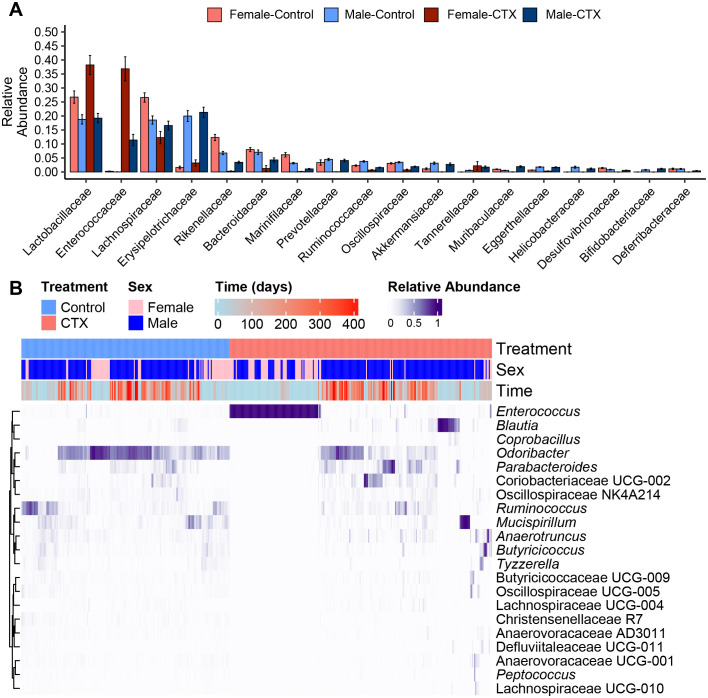
**(A)** Bar graph showing differentially abundant bacterial taxa at the family level across sex and treatment groups. **(B)** Heatmap showing the relative abundance of bacterial taxa across sex and treatment groups. Both panels are based on fecal 16S rRNA gene profiles from Control and ceftriaxone (CTX)-treated mice.

Across all samples, the most abundant bacterial families were Lactobacillaceae, Lachnospiraceae, and Erysipelotrichaceae (**Figure 3A**). Controlling for time, CTX treatment was associated with large and statistically significant shifts in family−level composition. Enterococcaceae showed the strongest treatment effect with a fold increase of 46.4 in CTX (q ≤ 1.5×10^−43^). Additional families enriched in CTX−treated mice included Peptostreptococcaceae, Tannerellaceae, Clostridiaceae, Atopobiaceae, Erysipelatoclostridiaceae, and Erysipelotrichaceae (fold differences = 4.34-31.4; q ≤ 2.4×10^−5^).

Families more abundant in The Control group included Bacteroidaceae, Acholeplasmataceae, Christensenellaceae, Rikenellaceae, Aerococcaceae, Defluviitaleaceae, Anaerovoracaceae, Marinifilaceae, Butyricicoccaceae, and Deferribacteraceae (fold differences = 2.97-6.75; q ≤ 1.0×10^−2^).

Sex was also associated with family−level differences. Families enriched in females included Lactobacillaceae, Enterobacteriaceae, Staphylococcaceae, Clostridiaceae, Rikenellaceae, and Marinifilaceae (fold differences = 2.86-9.99; q ≤ 3.3×10^−2^). Families enriched in males included Bifidobacteriaceae, Anaerofustaceae, Defluviitaleaceae, Monoglobaceae, Christensenellaceae, Akkermansiaceae, Erysipelotrichaceae, Deferribacteraceae, Acholeplasmataceae, and Muribaculaceae (fold differences = 2.75-206.0; q ≤ 1.1×10^−3^).

Controlling for time, CTX treatment was associated with significant shifts in genus−level composition (**Figure 3B**). *Enterococcus* showed the strongest treatment effect, with a 21.0-fold increase in the CTX group (q ≤ 1.6 × 10^−25^). Additional genera enriched in CTX−treated mice included *Coprobacillus*, *Blautia*, Coriobacteriaceae UCG−002, *Parabacteroides*, *Christensenella*, and *Turicibacter* (fold differences = 3.04-6.49; q ≤ 7.6×10^−3^).

Genera more abundant in Control mice included Oscillospiraceae NK4A214 group, Lachnospiraceae UCG−010, Anaerovoracaceae Family XIII AD3011 group, *Ruminococcus*, *Mucispirillum*, Anaerovoracaceae Family XIII UCG−001, *Tyzzerella*, *Butyricicoccus*, *Peptococcus*, and Christensenellaceae R−7 group (fold differences = 4.80-16.46; q ≤ 2.1×10^−5^).

Sex was also associated with genus−level differences, with female-enriched genera including *Parabacteroides*, *Ligilactobacillus*, *Escherichia*, *Enterococcus*, *Lactobacillus*, *Odoribacter*, HT002, *Bilophila*, Rikenellaceae RC9 group, and *Coprobacillus* (fold differences = 4.22-4.56×10^−15^; q ≤ 3.1×10^−3^). Genera more abundant in males included *Blautia*, *Butyricimonas*, *Bifidobacterium*, *Gordonibacter*, *Lachnoclostridium*, *Faecalibaculum*, *Anaerofustis*, Coriobacteriaceae UCG−002, *Parvibacter*, and *Akkermansia* (fold differences = 4.72-2.29×10^4^; q ≤ 2.2×10^−3^).

### Taxon-specific temporal dynamics following antibiotic treatment

**Figure 4:**
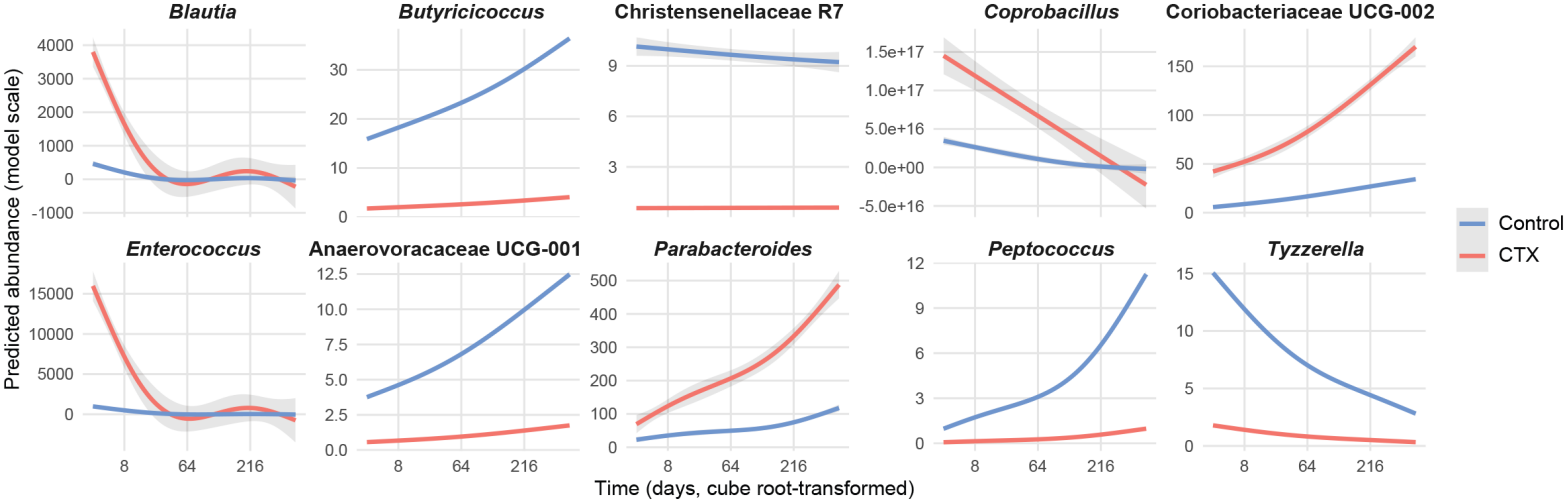
Predicted abundance trajectories of the top five positively and negatively differentially abundant bacterial taxa between Control and ceftriaxone (CTX) -treated mice over time. Each panel displays the fitted abundance (log-scale) of a single taxon, modeled while controlling for sex, treatment group, and time (cube-root transformed). Time 0 corresponds to the point of CTX cessation, with subsequent time points reflecting microbial recovery in CTX-treated mice and age-associated changes in Controls. Taxa were selected based on statistical significance (*q* < 0.01) and a minimum absolute fold difference of 2.0.

Following cessation of CTX treatment (Time 0), we examined temporal dynamics of top microbial taxa using predicted abundances from a Maaslin2 model controlling for Treatment, Sex, and Time (cube root-transformed continuous variable). Top features were selected based on a q-value cutoff of 0.01 and a fold-change threshold of 2.0, ensuring that only the most statistically significant and biologically relevant taxa were analyzed. The predicted abundances diverged between CTX-treated and Control mice over time, reflecting both microbiome recovery and age-associated changes (**Figure 4**). GAM smoothed curves illustrate the trajectories of each taxon while accounting for sex-specific and group-specific effects. *Blautia*, *Enterococcus*, and *Coprobacillus* were elevated in CTX mice immediately post-treatment but declined or stabilized at later timepoints. In Controls, these taxa remained low or showed gradual increases, consistent with age-related microbial succession rather than perturbation-driven expansion. In contrast, taxa such as *Peptococcus*, *Tyzzerella*, and Christensenellaceae R7 were suppressed in CTX-treated mice throughout the time course, while their abundance increased steadily in Controls, suggesting that antibiotic exposure disrupted their normal age-associated enrichment. *Butyricicoccus* increased over time in Controls but remained unchanged in CTX mice, whereas *Parabacteroides* showed modest increases in both groups, though with a higher initial abundance in CTX mice. These patterns highlight taxon-specific trajectories shaped by antibiotic exposure and natural aging, underscoring the long-term restructuring of the gut microbiome following treatment.

### Inferred pathway analysis

**Figure 5:**
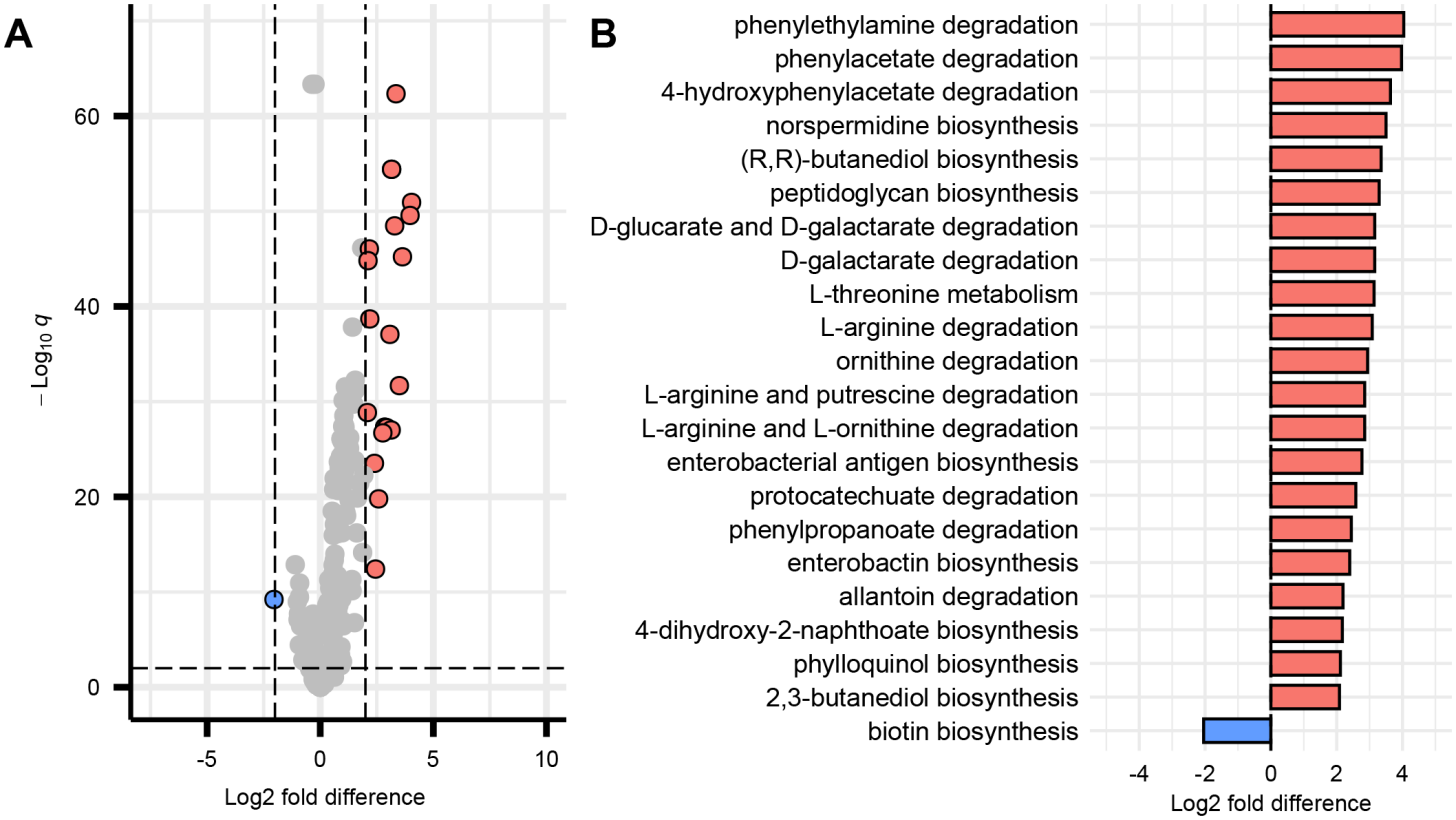
Differential abundance of inferred microbial pathways. **(A)** Volcano plot showing the differentially abundant microbial pathways between Control and ceftriaxone (CTX) -treated mice. **(B)** Horizontal bar chart showing log_2_ fold differences of significantly altered metabolic pathways between groups. Microbial pathways were inferred using PICRUSt2, and differential abundance was assessed with MaAsLin2 using a _q_-value cutoff of *q* < 0.01, |log_2_ fold difference| ≥ 2. Pathways enriched in Controls are shown in blue, those enriched in CTX are shown in red, and pathways not meeting either threshold are shown in gray.

Differential pathway analysis using PICRUSt2 revealed significant functional shifts in CTX-treated communities (**Figures 5A, B**). CTX exposure enriched pathways involved in aromatic compound degradation, including phenylethylamine, phenylacetate, 4-hydroxyphenylacetate, protocatechuate, and phenylpropanoate degradation (fold differences =5.4–16.4). Several amine and amino acid metabolism pathways were also elevated in response to CTX exposure, such as norspermidine biosynthesis, L-threonine metabolism, L-arginine and ornithine degradation, and combined L-arginine/putrescine or ornithine catabolism (fold differences =7.2–11.3). Additionally, CTX-treated communities showed enrichment of butanediol biosynthesis ((R,R)- and 2,3-butanediol), sugar acid degradation (D-galactarate and D-glucarate/D-galactarate), and peptidoglycan-related biosynthesis pathways, including peptidoglycan, enterobacterial antigen, allantoin, 1,4-dihydroxy-2-naphthoate, and phylloquinol (fold differences =4.3–9.8). In contrast, the only pathway enriched in Control samples was biotin biosynthesis (fold differences =4.1).

### Taxon-pathway associations

**Figure 6:**
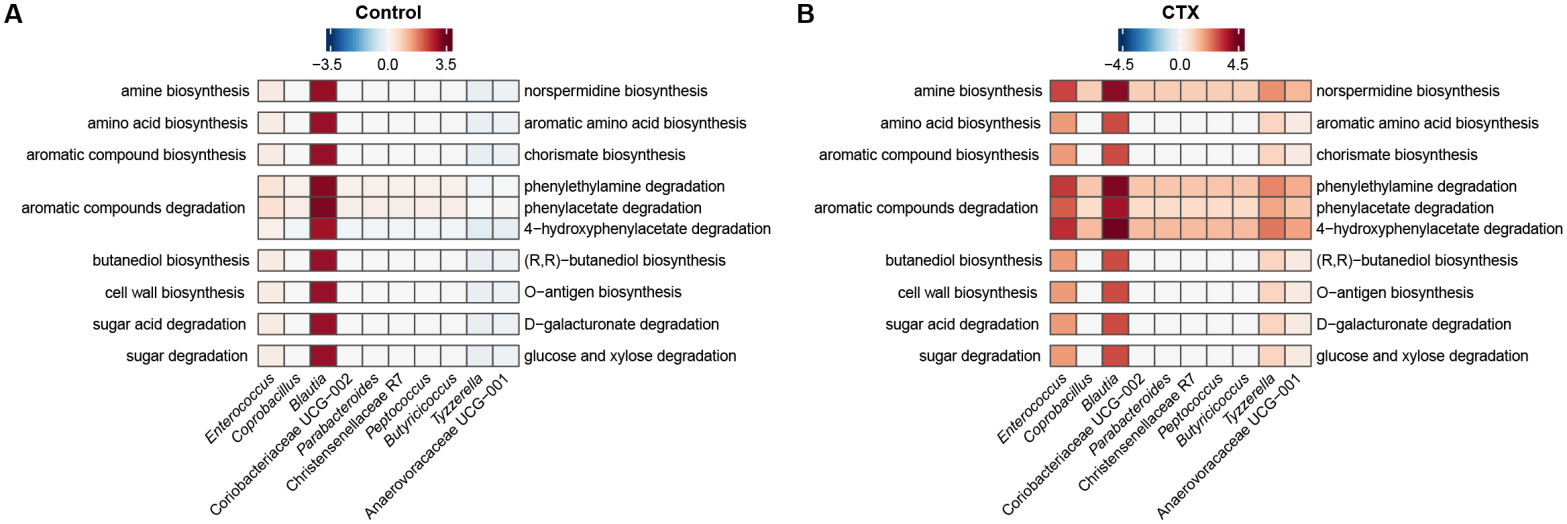
Associations between top bacterial taxa and microbial metabolic pathways in Control and ceftriaxone (CTX)-treated mice. Heatmaps show posterior mean associations between top differentially abundant bacterial taxa (columns) and top microbial pathways (rows) in Control **(A)** and CTX-treated mice **(B)**. Taxa were selected based on differential abundance between CTX-treated and Control mice (MaAsLin2 *q* < 0.01, |log_2_ fold difference| ≥ 2). Pathways were similarly selected and grouped by functional category, indicated on the left. Colors represent the magnitude of posterior mean association, with red indicating positive and blue indicating negative values. Columns and rows are matched across panels to facilitate comparison of treatment-specific remodeling of taxon-pathway associations.

Analysis of taxon-pathway associations revealed treatment-specific remodeling of microbial functional connectivity, with certain taxa showing strengthened correlations with key metabolic pathways in CTX-treated mice compared with controls. Heatmaps (**Figure 6**) display taxon-pathway associations for which BZINB regression identified significant effects (q < 0.01, |log2 fold-change| ≥ 1), illustrating treatment-specific changes in functional connectivity. In control mice, *Blautia* exhibited broad positive associations across multiple anabolic and catabolic pathways (9 strong associations; total connectivity 19.1), whereas *Enterococcus* showed weaker associations (2 strong associations; total connectivity 7.9) and other taxa displayed minimal connectivity.

In CTX-treated mice, several associations strengthened. The ten strongest CTX-strengthened taxon-pathway pairs involved *Enterococcus* (total connectivity shift 19.7), Anaerovoracaceae UCG-001 (14.1), and *Tyzzerella* (13.8), with pathways largely related to aromatic compound degradation and amine biosynthesis. Categorization of these strengthened associations confirmed the predominance of aromatic compound degradation (seven pairs) and amine biosynthesis (three pairs). Hub pathways strengthened across ≥2 taxa were 4-hydroxyphenylacetate degradation, norspermidine biosynthesis, and phenylethylamine degradation.

## Discussion

### Findings of the current study

Across four independent murine experiments, CTX exposure produced robust changes in overall diversity metrics and in the relative abundances of specific taxa, alongside shifts in predicted microbial function. CTX treatment reduced alpha diversity and shifted beta diversity, with clear separation between CTX and Control communities and partial convergence over time; both sex and time contributed independently to community variation, and males showed a steeper recovery in richness. CTX-treated mice exhibited characteristic increases in several antibiotic−responsive genera, including *Enterococcus* and *Coprobacillus*, and decreases in taxa more abundant in Controls, such as *Ruminococcus* and *Mucispirillum*, while sex contributed additional differences in taxonomic profiles, including higher relative abundance of *Bifidobacterium* in males and *Enterococcus* in females. Functional predictions indicated broad metabolic shifts following CTX exposure, with increased representation of pathways involved in aromatic compound degradation, amine metabolism, sugar−acid degradation, and peptidoglycan−related biosynthesis, whereas Control-related communities retained higher predicted biotin biosynthesis. Bayesian modeling further showed treatment−specific restructuring of taxon-pathway connectivity, with Control mice displaying diffuse, low−magnitude associations across many taxa and CTX-treated mice showing stronger, more concentrated associations driven by a smaller subset of genera across aromatic compound degradation and related metabolic pathways.

### Findings of the current study in context of previous studies

Our findings are consistent with prior work showing that broad−spectrum antibiotics, including several β−lactams, can reduce microbial diversity and alter gut community structure in mice (Gu et al., 2020; Hertz et al., 2020; Gao et al., 2024). Several commensal taxa, including *Butyricicoccus*, Christensenellaceae R7, and *Ruminococcus*, showed slow or incomplete recovery following CTX exposure, whereas opportunistic taxa such as *Enterococcus*, *Tyzzerella*, and *Parabacteroides* expanded. These outcomes mirror previous reports of persistent commensal depletion coupled with opportunist enrichment after antibiotic treatment (Ng et al., 2019; Ramirez et al., 2020; Fishbein et al., 2023; Cusumano et al., 2025), underscoring the marked sensitivity of key commensals and the ecological resilience of opportunistic taxa during microbiome disturbance.

By integrating taxonomic shifts with predicted metabolic functions, this cross−study analysis shows that CTX exposure restructures not only community composition but also microbial functional potential. *Enterococcus* exhibited strengthened associations with aromatic compound and amine metabolism, while *Blautia* maintained broad connectivity with both anabolic and catabolic pathways across conditions. These findings indicate that CTX−driven compositional changes are accompanied by selective reinforcement of metabolic pathways in taxa adapted to the post−antibiotic environment, consistent with prior observations of antibiotic−associated functional remodeling in mice, rats, and humans (Francino, 2015; Behr et al., 2017; He et al., 2023; Wang et al., 2024).

Predicted functional changes with CTX exposure indicated increased representation of pathways involved in aromatic compound and amine metabolism, amino acid catabolism, and stress−associated biosynthesis, many of which generate metabolites known to influence epithelial barrier function, immune signaling, and neuromodulatory processes (Zelante et al., 2013; Rooks and Garrett, 2016; Roager and Licht, 2018; Strandwitz, 2018; Cryan et al., 2019). Reduced predicted biotin biosynthesis in CTX−treated communities suggests diminished microbial contributions to micronutrient availability, with potential implications for host metabolic and neuronal processes (Zempleni et al., 2008; Magnúsdóttir et al., 2015; Sedel et al., 2015; Mock, 2017). Strengthened associations between *Enterococcus* and *Tyzzerella* and aromatic or amine metabolism further suggest that CTX−driven niche restructuring may favor taxa capable of producing metabolites relevant to gut-brain communication (Abdugheni et al., 2022; Rahmdel et al., 2025), while the stability of *Blautia*’s metabolic associations may reflect a conserved role in maintaining baseline microbial metabolic activity.

This study provides the first robust, cross-study assessment of CTX’s impact on inferred microbial functions in mice, enabling systematic evaluation of persistent and treatment-specific functional alterations. Together, these results reveal a treatment-specific reconfiguration of the murine microbiome’s metabolic network, highlighting both the resilience and vulnerability of microbial functions following CTX exposure. The integration of taxonomic and functional perspectives clarifies how CTX reshapes microbial metabolic connectivity, identifying both reinforced pathways in resilient taxa and diminished contributions from depleted commensals.

Although some time points did not include both sexes, several outcomes exhibited sex−associated differences, including model−estimated temporal recovery patterns in which males showed a steeper increase in richness following CTX exposure. Modeled taxonomic profiles also suggested sex−linked differences in community composition, with higher relative abundance of *Bifidobacterium* in males and *Enterococcus* in females. These patterns align with prior work showing that antibiotic responses can vary by sex due to differences in immune tone, hormone-microbiome interactions, and baseline microbial composition (Markle et al., 2013; Gao et al., 2019; Kim et al., 2020). The faster recovery in males, together with sex−biased shifts in specific taxa, may reflect sex−dependent differences in mucosal immune activity, epithelial turnover, or microbial metabolic capacity that influence recolonization dynamics after β−lactam exposure. Collectively, these findings indicate that CTX−induced restructuring of microbial composition and function varies across hosts, underscoring the importance of incorporating sex as a biological variable in future antibiotic-microbiome studies.-.

Beyond these sex−associated patterns, CTX also has documented neurophysiological effects. CTX is known to increase GLT−1 expression (Rothstein et al., 1996; Rothstein et al., 2005), and antibiotic−induced microbiome perturbations can influence neurotransmitter−related pathways through altered precursor metabolism, neuroactive compound production, and neuroimmune signaling routes (Strandwitz, 2018; Baj et al., 2019; Angoa-Pérez and Kuhn, 2021; Chen et al., 2021; Margolis et al., 2021; Swer et al., 2023; Loh et al., 2024; Mhanna et al., 2024). Persistent microbial alterations following CTX exposure may therefore contribute to changes in neuroactive metabolite availability and gut-brain signaling (Camberos-Barraza et al., 2024; Dezfouli et al., 2024; Lee et al., 2024; Kern et al., 2025; Xu and Lu, 2025), providing a framework for understanding how CTX−induced remodeling of microbial composition and function could have neurophysiological effects on barrier integrity, immune signaling, and metabolic homeostasis (Holota et al., 2019).

## Limitations

Several methodological considerations should be noted. Sample sizes across the four reanalyzed datasets were generally insufficient to robustly evaluate the influence of mouse strain, sex, antibiotic dose, or route of administration on microbiome outcomes. One dataset (Grégoire et al., 2021) included a single gavage of *Klebsiella pneumoniae* CTX-M9 during antibiotic treatment (Grégoire et al., 2021). Although this feature introduced an additional experimental variable, the dataset was retained to increase overall sample size and to incorporate longitudinal sampling with appropriate controls, thereby strengthening the temporal scope of the re-analysis.

Technical factors may also have influenced observed patterns. Differences in 16S rRNA sequencing platforms and targeted variable regions across studies could affect taxonomic resolution and relative abundance estimates. The included studies differ in duration, sampling windows, and sequencing protocols, resulting in some heterogeneity in multivariate dispersion despite standardized processing; however, sensitivity analyses indicate that these differences did not alter the observed CTX-associated community shifts. In addition, functional predictions generated using PICRUSt2 represent inferred metabolic potential based on reference genomes rather than direct measurements of microbial gene expression or metabolite production.

Multiple computational approaches have been developed for the integration of microbiome datasets across studies. For example, MMUPHin is a Bioconductor framework that provides tools for covariate-adjusted batch correction, differential abundance testing, and identification of shared microbial community structures across datasets (Ma et al., 2022). This represents one of several available analytical strategies and was not applied in the present study. Our approach enabled the identification of microbial and functional patterns that were reproducible across independent datasets while minimizing methodological variability, which aligned with the goals of this study.Despite these limitations, applying a standardized analytical workflow across all datasets enabled the identification of consistent taxonomic and functional patterns following CTX exposure. Temporal analyses, combined with modeling of bacterial taxa-metabolic associations, further allowed quantification of taxon-specific functional linkages, providing mechanistic insight into community restructuring and clarifying the broader ecological and metabolic consequences of antibiotic-induced dysbiosis.

## Future directions and translational implications

Although zero-inflated negative binomial models provide a flexible framework for handling sparsity in microbiome data, recent benchmarking studies have shown that alternative approaches such as ANCOMBC2 and other zero-inflated or bias-corrected methods can also perform well under different conditions (Yang and Chen, 2022; Lin and Peddada, 2024). Future work could compare these frameworks directly in the context of taxon-pathway association analysis.

Future studies should validate predicted metabolic shifts and establish causal links between microbiome alterations and host physiology using targeted metabolomics and multi-omics approaches. Experimental interventions, such as dietary modulation or prebiotic/probiotic treatments, alongside longitudinal monitoring, could determine whether microbial recovery and functional restoration can be accelerated. Integrating microbiome profiling with host measurements, including gut barrier function, immune responses, or neuroimmune signaling, would help evaluate potential associations between microbial recovery and downstream physiological outcomes. From a translational standpoint, defining these links could support the development of microbiome−informed biomarkers of antibiotic impact, identify individuals who may benefit from targeted microbial support, and guide strategies aimed at restoring metabolic functions relevant to gut, immune, or gut-brain signaling pathways. More broadly, this work highlights the value of standardized cross-study meta-analyses for identifying robust patterns across diverse datasets.

## Conclusions

This re-analysis of murine CTX studies demonstrates that broad-spectrum antibiotic exposure consistently perturbs the gut microbiome, inducing long-lasting, taxon-specific changes in microbial community composition and functional potential. These alterations emphasize both the ecological and metabolic consequences of CTX treatment, including shifts in microbial functional connectivity and resilience. Our findings highlight the value of standardized, cross-study bioinformatic approaches for identifying reproducible microbial signatures and provide a framework for future mechanistic investigations into how antibiotic-induced remodeling of the gut microbiome can have drastic effects on host physiology.

## Data Availability

The original contributions presented in the study are included in the article/**Supplementary Material**. Further inquiries can be directed to the corresponding author.
